# Inflammatory Conditions Induce IRES-Dependent Translation of cyp24a1

**DOI:** 10.1371/journal.pone.0085314

**Published:** 2014-01-08

**Authors:** Daniela Rübsamen, Michael M. Kunze, Victoria Buderus, Thilo F. Brauß, Magdalena M. Bajer, Bernhard Brüne, Tobias Schmid

**Affiliations:** Institute of Biochemistry I, Faculty of Medicine, Goethe-University Frankfurt, Frankfurt, Germany; German Cancer Research Center, Germany

## Abstract

Rapid alterations in protein expression are commonly regulated by adjusting translation. In addition to cap-dependent translation, which is e.g. induced by pro-proliferative signaling via the mammalian target of rapamycin (mTOR)-kinase, alternative modes of translation, such as internal ribosome entry site (IRES)-dependent translation, are often enhanced under stress conditions, even if cap-dependent translation is attenuated. Common stress stimuli comprise nutrient deprivation, hypoxia, but also inflammatory signals supplied by infiltrating immune cells. Yet, the impact of inflammatory microenvironments on translation in tumor cells still remains largely elusive. In the present study, we aimed at identifying translationally deregulated targets in tumor cells under inflammatory conditions. Using polysome profiling and microarray analysis, we identified cyp24a1 (1,25-dihydroxyvitamin D_3_ 24-hydroxylase) to be translationally upregulated in breast tumor cells co-cultured with conditioned medium of activated monocyte-derived macrophages (CM). Using bicistronic reporter assays, we identified and validated an IRES within the 5′ untranslated region (5′UTR) of cyp24a1, which enhances translation of cyp24a1 upon CM treatment. Furthermore, IRES-dependent translation of cyp24a1 by CM was sensitive to phosphatidyl-inositol-3-kinase (PI3K) inhibition, while constitutive activation of Akt sufficed to induce its IRES activity. Our data provide evidence that cyp24a1 expression is translationally regulated via an IRES element, which is responsive to an inflammatory environment. Considering the negative feedback impact of cyp24a1 on the vitamin D responses, the identification of a novel, translational mechanism of cyp24a1 regulation might open new possibilities to overcome the current limitations of vitamin D as tumor therapeutic option.

## Introduction

The 5′ untranslated region (5′UTR) of mRNAs is important for translation initiating events as the translation initiation machinery assembles here to recruit ribosomes [1]. Since the initiation step constitutes the primary level of regulation of translation, the formation of the initiation complex, comprising eukaryotic initiation factors (eIFs) such as the RNA helicase eIF4A, the scaffolding protein eIF4G, and the cap-binding protein eIF4E is highly regulated [2]. The mammalian target of rapamycin (mTOR) kinase was identified as a key regulator of translation initiation. Specifically, mTOR activates p70^S6K^ by phosphorylation, which in turn phosphorylates the 40S ribosomal subunit [3]. In addition, mTOR inhibits the 4E-binding protein (4E-BP), which upon mTOR-dependent hyperphosphorylation releases the cap-binding protein eIF4E, thus allowing for initiation of cap-dependent translation [4], [5]. Enhanced activation of phosphatidyl-inositol-3-kinase (PI3K)-mTOR signaling, which is commonly observed in tumors, stimulates the translation of various tumor-associated factors with highly structured 5′UTRs such as cyclin D1 [6]. Deregulated translation is therefore increasingly appreciated as a target for the development of tumor therapeutics, yet translation-oriented therapies (e.g. rapamycin and its analogues) so far were focused mainly on the inhibition of mTOR [7], [8]. Importantly, the protein synthesis of various survival factors is maintained in a cap-independent manner, e.g. via internal ribosome entry sites (IRES), under conditions where cap-dependent translation is impaired [9], [10]. IRES elements facilitate initiation of translation independently of the cap-binding protein eIF4E and were described for oncogenes like the hypoxia-inducible factor 1α [11], the inhibitor of apoptosis proteins [12], and b-cell lymphoma 2 [13]. Activation of IRES elements commonly requires the presence and/or activity of so-called IRES *trans*-acting factors (ITAFs) such as the polypyrimidine tract binding protein [14]. Binding of these proteins was proposed to initiate conformational changes of the 5′UTR structure, thereby facilitating the interaction of eIFs and ribosomal subunits with the mRNA.

While there is mounting evidence that translational deregulation plays an important role in tumorigenesis, the impact of altered translation during inflammation-driven tumorigenesis remains elusive. Yet, mediators within the inflammatory environment such as members of the interleukin-1 family have been shown to control protein expression on a translational level [15], [16]. The present study aimed at characterizing a novel, translationally regulated target during inflammation-associated tumorigenesis. Specifically, cyp24a1 (1,25-dihydroxyvitamin D_3_ 24-hydroxylase) was translationally upregulated in breast tumor cells treated with supernatants of activated monocyte-derived macrophages using polysome profiling and microarray analysis. Our data revealed the presence of an IRES element within the 5′UTR of cyp24a1 that was PI3K-Akt-dependently induced by inflammatory conditions.

## Materials and Methods

### Materials

All chemicals were purchased from Sigma-Aldrich (Schnelldorf, Germany), if not indicated otherwise. Rapamycin came from Cell Signaling Technology (Frankfurt, Germany), LY294002 and SB203580 from Enzo Life Science (Lörrach, Germany). Antibodies were obtained from the following sources: anti-Akt and anti-phospho-S6 from Cell Signaling Technology (Frankfurt, Germany), anti-HA from Covance (Munich, Germany), anti-nucleolin from Santa Cruz Biotechnology (Heidelberg, Germany), and IRDyes 680LT and 800CW secondary antibodies from Li-COR Biosciences GmbH (Bad Homburg, Germany).

### Cell culture

Cell lines came from LGC Standards GmbH (Wesel, Germany). MCF7 and U937 cells were maintained in RPMI medium supplemented with 10% fetal bovine serum (FBS), 100 U/mL penicillin, 100 µg/mL streptomycin, and 2 mM L-glutamine. Additionally, U937 media contained 1 mM sodium pyruvate. HEK293 cells stably expressing constitutively active myr Akt were created via retroviral transduction and have been previously described [17]. Stable HEK293 cells were maintained in DMEM medium containing 10% FBS, 100 U/mL penicillin, 100 µg/mL streptomycin, 2 mM L-glutamine, 110 mg/L sodium pyruvate, and 1 mg/mL G418 for selection purposes. All cells were kept at 37°C in a humidified atmosphere with 5% CO_2_. Medium and supplements were purchased from PAA (Linz, Austria). FBS came from Biochrom (Berlin, Germany).

### Macrophage differentiation and conditioned medium

U937 monocytes (1×10^7^/25 mL) were exposed to 10 nM 12-*O*-tetradecanolyphorbol-13-acetate (TPA) for 48 h. The resulting adherent and activated U937 monocyte-derived macrophages were trypsinized, pelleted and washed with PBS. For control purposes undifferentiated U937 monocytes (3×10^6^/25 mL) were incubated with DMSO (0.1%) for 48 h, pelleted by centrifugation and washed with PBS. Subsequently, control and differentiated U937 were treated equally. For the generation of conditioned medium U937 cells were reseeded at a concentration of 2×10^6^/5 mL and allowed to condition medium for 24 h. Conditioned medium was harvested by centrifugation and sterile filtration (0.45 µm filter), and subsequently stored at -80°C until further use. All experiments were carried out in U937 medium.

### Polysomal fractionation

5×10^6^ MCF7 cells were seeded in a 15 cm dish 1 day prior to treatment of the cells followed by polysomal fractionation. Briefly, after incubation with 100 µg/mL cycloheximide (CHX) for 10 min at 37°C, cells were harvested in PBS/CHX [100 µg/mL] and lysed in 750 µL polysome buffer [140 mM KCl, 20 mM Tris-HCl (pH 8.0), 5 mM MgCl_2_, 0.5% NP40, 0.5 mg/mL heparin, 1 mM DTT, 100 U/mL RNasin (Promega, Mannheim, Germany), 100 µg/mL CHX]. After pelleting, the cytoplasmic lysates were layered onto 11 mL 10–50% continuous sucrose gradients. The gradients were centrifuged at 35000 rpm for 2 h at 4°C without brake using a SW40 rotor in a Beckman ultracentrifuge. Afterwards the gradients were collected in 1 mL fractions using a Biologic LP system (Biorad, München, Germany). Absorbance was measured at 254 nm. RNA was precipitated by 1/10 volume sodium acetate [3 M] and 1 volume isopropanol. RNA was further purified using the RNeasy MiniKit (Qiagen) according to the manufacturer's manual. For quality control, equal volumes of RNA of each fraction were analyzed by denaturing agarose gel electrophoresis. RNA was transcribed using the Maxima First Strand cDNA synthesis kit from Thermo Fisher (St. Leon-Rot, Germany) and subsequently individual mRNAs were analyzed using realtime PCR with iQ SybrGreen Supermix (Biorad, München, Germany). Results are shown as percentage of mRNA in the single fractions relative to the total amount of mRNA extracted from all fractions. Specific primers were individually designed (gapdh-fwd: TGC ACC ACC AAC TGC TTA GC, gapdh-rev: GGC ATG GAC TGT GGT CAT GAG; cyp24a1-fwd: AGC TTC AAC TGC ATT TGG CT, cyp24a1-rev: AAA TAC CAC CAT CTG AGG CG; *firefly*-fwd: ATT TAT CGG AGT TGC AGT TGC GCC, *firefly*-rev: GCT GCG AAA TGC CCA TAC TGT TGA, *renilla*-fwd: CAG TGG TGG GCC AGA TGT AAA CAA, *renilla*-rev: TAA GAA GAG GCC GCG TTA CCA TGT).

### Plasmid construction

Primers were designed to amplify the human cyp24a1-5′UTR using RNA extracted from human MCF7 cells (fwd: CAT ACT AGT GAC AGG AGG AAA CGC AGC GCC AGC AG, rev: ATC CAT GGT CCT GCC TTC CCG CGC TC). A single product of 398 nucleotides was obtained and inserted into the hairpin-containing bicistronic vector phpRF (kind gift of Prof. Anne Willis [18]) with SpeI and NcoI resulting in phpR-cyp-F. To rule out cryptic splicing events mediated by the 5′UTR of cyp24a1 in the bicistronic vectors, primers were designed to bind to the 5′end of the *renilla* ORF and the 3′end of the *firefly* ORF (fwd: ATG ACT TCG AAA GTT TAT GAT CCA GAA CAA AGG AAA CGG, rev: TTA CAC GGC GAT CTT TCC GCC CT).

### Reporter assays

MCF7 cells were transiently transfected with 0.2 µg DNA using Rotifect reagent (Roth, Karlsruhe, Germany) according to the manufacturer's protocol. After 16 h, medium was changed and cells were stimulated. Following stimulation, cells were lysed and *firefly* and *renilla* luciferase activities were determined using a Dual Luciferase kit assay (Promega) on a Mithras LB 940 luminometer (Berthold, Bad Wildbad, Germany).

For RNA transfections the DNA constructs were linearized with *BamHI*, *in vitro*-transcribed using the mMESSAGE mMACHINE T7 Kit (Ambion) according to the manufacturer's protocol, and purified with the MEGAclear Kit (Ambion). 0.2 µg RNA were transfected as described for DNA. Luciferase activities were measured 24 h post transfection.

### Western analysis

For Western analysis, cells were sonicated and then lysed on ice for 30 min in lysis buffer [50 mM Tris–HCl, 150 mM NaCl, 5 mM ethylenediaminetetraacetic acid, 0.5% NP-40, 1 mM phenylmethylsulfonyl fluoride and protease inhibitor mix (Roche, Mannheim, Germany)]. 50 µg protein were separated on SDS-polyacrylamide-gels and transferred onto nitrocellulose membranes. Proteins were detected using specific antibodies and appropriate secondary antibodies and visualized on an Odyssey infrared imaging system (Li-COR Biosciences GmbH, Bad Homburg, Germany).

### Statistical analysis

Each experiment was performed at least three times. Data are presented as means ± SEM. Statistical analysis was performed using Student's t-test.

## Results

### Cyp24a1 translation is induced under inflammatory conditions

**Figure 1:**
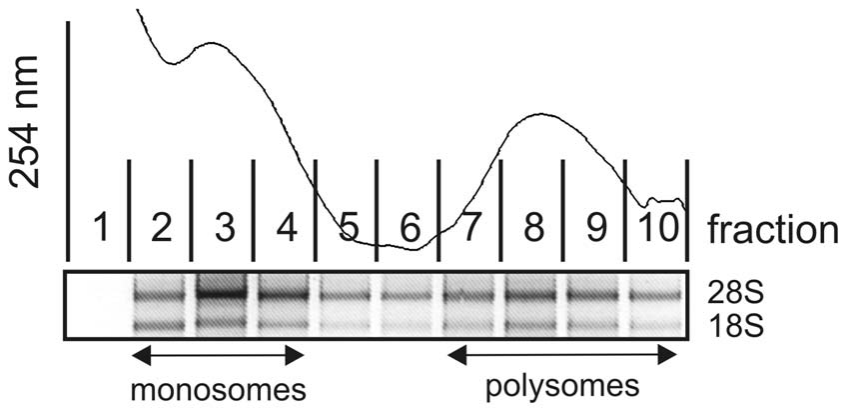
Polysome profile of MCF7 cells. Representative profile of MCF7 lysates at 254 nm as determined during polysomal fractionation (*upper panel*). Equal aliquots of RNA isolated from single fractions were analyzed using denaturing agarose gel electrophoresis to verify 28S and 18S rRNA content as indicators for ribosome distribution (*lower panel*).

We have previously established that supernatants of TPA-activated U937 monocyte-derived macrophages (CM) contain elevated levels of pro-inflammatory mediators as compared to control supernatants of undifferentiated U937 cells (Ctr). CM further induced transformation and migration of MCF7 mammary carcinoma cells [19], [20]. Importantly, we noticed that such inflammatory conditions induce translational changes in MCF7 cells as determined by altered polysomal association of various mRNAs [16]. Polysomal fractionation analyses of MCF7 cells (Figure 1) followed by microarray analyses of polysomal mRNA changes in response to 4 h CM treatment [16], identified cyp24a1 to significantly increase in the polysomal fractions without concomitant change on total RNA level in response to 4 h CM treatment. Since cyp24a1 has been proposed to play a critical role in the development of resistances to tumor therapeutic application of vitamin D_3_, we aimed at further characterizing the translational regulation of cyp24a1.

**Figure 2:**
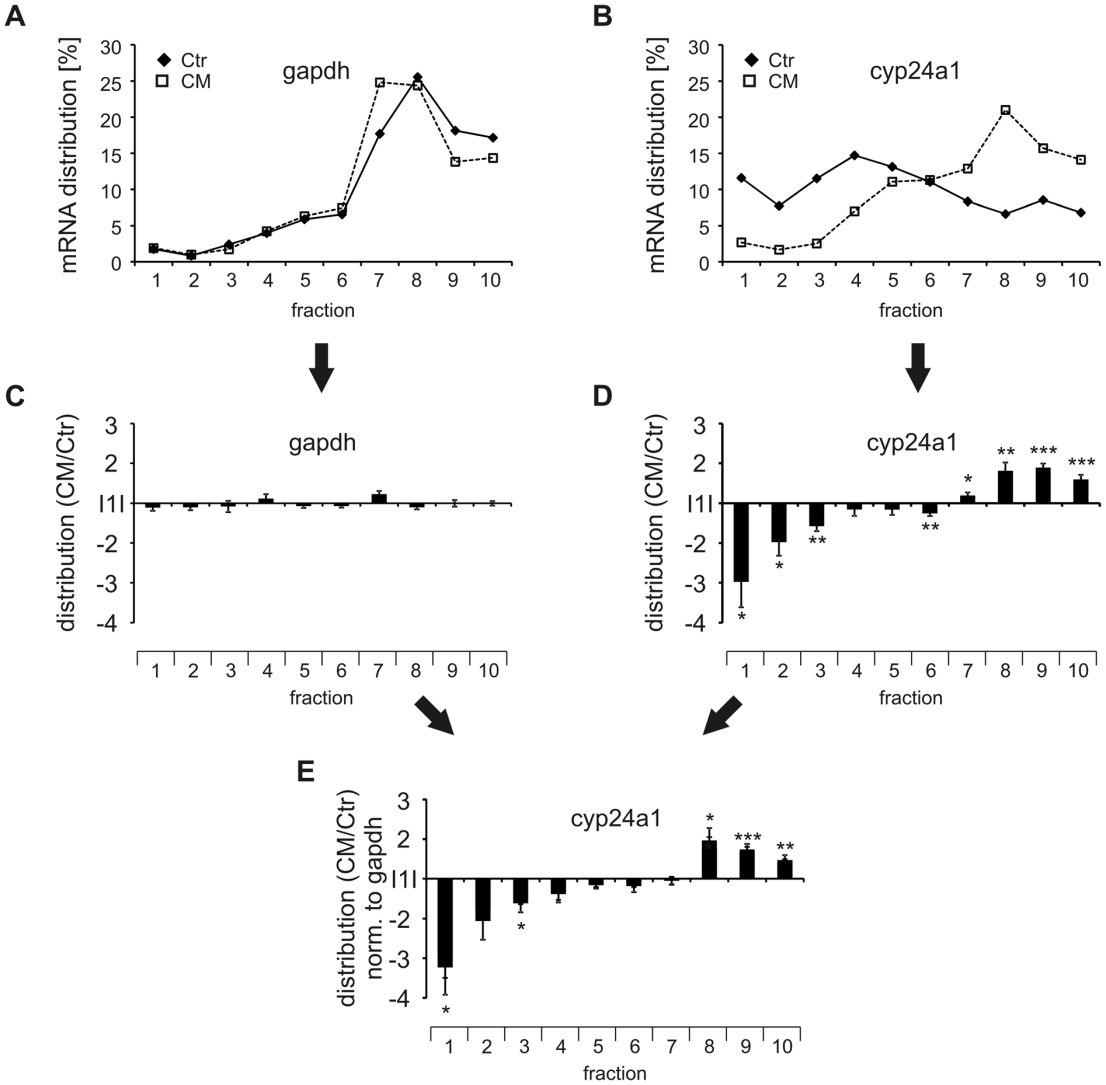
CM induces cyp24a1 translation. MCF7 cells were treated with Ctr or CM for 4(A) and cyp24a1 (B) was analyzed in single fractions using RT-qPCR. The distribution of the respective mRNAs across the individual gradients was determined relative to the total RNA extracted from the gradients. Results from a representative experiment are given in A and B. (C+D) Changes of gapdh (C) and cyp24a1 (D) mRNA distribution induced by CM were normalized to Ctr. (E) cyp24a1 distribution (from D) was normalized to gapdh distribution (from C). Distribution changes are presented as means ± SEM (n≥3, * p<0.05, ** p<0.01, *** p<0.001).

**Figure 3:**
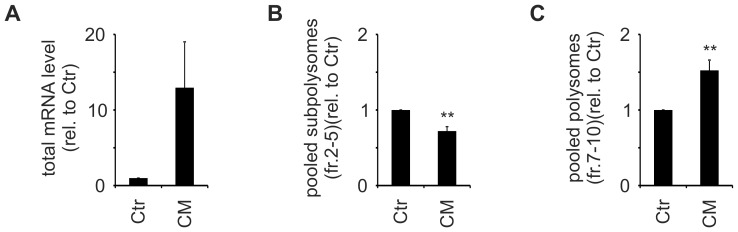
CM induces cyp24a1 mRNA expression and translation. MCF7 cells were treated with Ctr or CM for 4(A) Cyp24a1 mRNA expression changes were determined in total mRNA relative to gapdh. (B+C) The distribution changes across from Figure 2E were re-analyzed for the pooled subpolysomes (fract. 2–5) (B) and the pooled polysomes (fract. 7–10) (C) as indicators for translational regulation. Data are presented as means ± SEM (n≥3).

To verify that the translation of cyp24a1 indeed is enhanced by CM, we analyzed the relative abundance of cyp24a1 mRNA in single fractions after 4 h Ctr or CM treatment of MCF7 cells. As anticipated glyceraldehyde 3-phosphate dehydrogenase (gapdh) mRNA distribution appeared unaltered in response to CM (Figure 2A). In contrast, cyp24a1 mRNA distribution decreased in the monosomal and increased in the polysomal fraction in response to CM (Figure 2B). In order to obtain statistical information for these changes, we normalized the mRNA distributions in response to CM to the Ctr distribution for individual experiments and determined mean values for at least 3 independent experiments. Since no differences were observed for gapdh distribution (Figure 2C), but significant changes in the cyp24a1 distribution occurred (Figure 2D), gapdh was used to normalize cyp24A1 distribution (Figure 2E). In line with the unaltered distribution of gapdh mRNA, normalization did not change the distribution of cyp24a1 across the polysome profile, showing a decrease in sub-polysomal fractions and an increase in polysomal fractions in response to CM (Figure 2E). Surprisingly, CM also caused a marked, but variable increase in total cyp24a1 mRNA expression (12.95±6.06 compared to Ctr) (Figure 3A). Nevertheless, as cyp24a1 mRNA significantly decreased in the pooled subpolysomal fractions (0.72±0.06 of Ctr) (Figure 3B) and at the same time significantly increased in the pooled polysomal fractions (1.53±0.41 fold of Ctr) (Figure 3C), altered translation appeared to occur in parallel to mRNA expression changes. The observation that the polysomal redistribution appeared completely independent of the mRNA expression change across the different experiments further supports the notion of two distinct mechanisms affecting mRNA expression and translation regulation. Interestingly, the CM-enhanced polysomal association of cyp24a1 mRNA was similar to that previously observed for egr2 (1.96±0.13 fold) [16]. Thus, inflammatory conditions apparently induce the translation of cyp24a1, resulting in a specific enrichment of cyp24a1 mRNA in the late polysomes, i.e. highly translated fractions.

### Cyp24a1 translation can be regulated in a cap-independent manner

**Figure 4:**
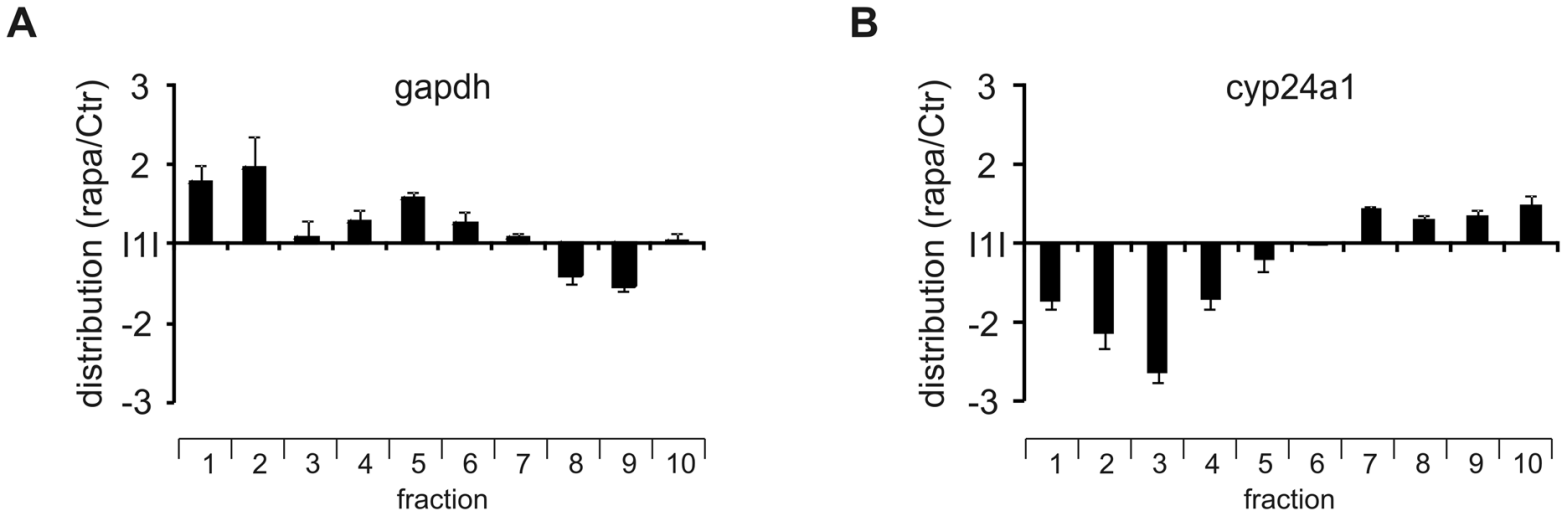
Cyp24a1 translation is initiated in part cap-independently. MCF7 cells were treated with rapamycin [100 nM] for 4 h and subjected to polysomal fractionation. RNA from single fractions was isolated and gapdh (A) and cyp24a1 (B) mRNA distribution changes were analyzed separately as described before. Data are presented as means ± SEM (n≥3).

As the 5′UTR of cyp24a1 mRNA is relatively long, spanning 399 nucleotides and *in silico* structural predictions using RNAfold [21] suggested a minimum free energy ΔG = −109.5 kcal/mol, where minimum free energies ΔG<−50 kcal/mol are considered to prevent effective scanning and translation initiation [22], we next tested whether cyp24a1 translation occurs in a cap-dependent or -independent manner. In line with re-activation of 4E-BP and concomitant attenuation of cap-dependent translation by mTOR inhibitors [23], treatment of MCF7 cells with 100 nM rapamycin for 4 h shifted gapdh mRNA distribution towards the sub-polysomal fractions (Figure 4A). Oppositely to gapdh, cyp24a1 mRNA moved from the sub-polysomal to the polysomal fractions upon rapamycin treatment (Figure 4B). Since gapdh mRNA distribution was altered by rapamycin, cyp24a1 mRNA distribution changes were not normalized to gapdh.

Taken together, the structured 5′UTR and the translational behavior of cyp24a1 towards rapamycin are indicative for cap-independent, i.e. IRES-dependent, cyp24a1 translation.

### Cyp24a1 contains an IRES element

**Figure 5:**
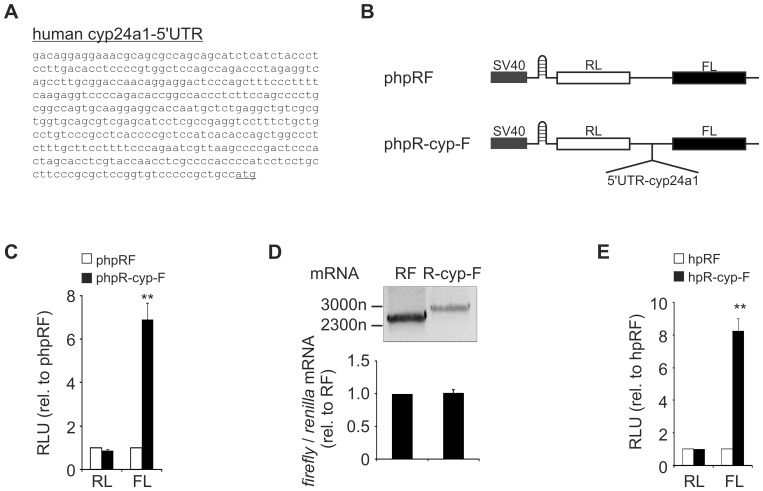
Cyp24a1 contains an IRES element. (A) Sequence of the human cyp24a1-5′UTR. (B) Schematic representation of the bicistronic control (phpRF) and cyp24a1-5′UTR-containing (phpR-cyp-F) luciferase constructs used for reporter assays. (C) Bicistronic reporter plasmids phpRF (white bars) and phpR-cyp-F (black bars) were transfected into MCF7 cells. 24 h after transfection *renilla* and *firefly* luciferase activities were measured and data are presented as means ± SEM relative to phpRF (n≥3, ** p<0.01). (D) RNA isolated from cells transfected with phpRF or phpR-cyp-F was DNAse treated and reverse transcribed. *Upper panel*: PCR was performed with specific primers to amplify full length RL or R-cyp-L mRNAs. PCR products were visualized *via* agarose gel electrophoresis and ethidium bromide staining. Data are representative for at least 3 independent experiments. *Lower panel*: RT-qPCR analysis of the amount of *firefly* mRNA normalized to *renilla* mRNA. Data are presented as means ± SEM (n≥3). (E) *In vitro-*transcribed mRNAs of the control (hpRF, white bars) or the cyp24a1-5′UTR-containing vector (hpR-cyp-F, black bars) were transfected into MCF7 cells. 24 h after transfection *renilla* and *firefly* luciferase activities were measured. Luciferase activities are given relative to hpRF and data are presented as means ± SEM (n≥3, ** p<0.01).

To prove IRES-dependent translation of cyp24a1, the 5′UTR of cyp24a1 (Figure 5A) was inserted into the bicistronic reporter vector phpRF. In this vector *renilla* luciferase is cap-dependently expressed at a low level due to a hairpin-structure. *Firefly* luciferase is only translated, if a sequence is inserted intercistronically that allows for alternative initiation, i.e. if a sequence containing an IRES element is introduced (Figure 5B) [18]. Both the cyp24a1-5′UTR containing phpR-cyp-F and the control phpRF vectors yielded similar levels of *renilla* luciferase signals when transfected into MCF7 cells. In contrast, the *firefly* luciferase significantly increased 6.93±0.75 fold upon insertion of the cyp24a1-5′UTR (Figure 5C). This increase is similar to that previously described for egr2 (7.12±0.61 fold compared to phpRF) [16] and strongly indicates the presence of an IRES element. Yet, the intercistronic insertion of the 5′UTR into the bicistronic vectors might result in cryptic splicing or promoter activities and consequently erroneous assignment of an IRES element [24]. To exclude the contribution of such cryptic events to the observed IRES activity, DNA-free RNA was isolated from MCF7 cells transfected with phpRF or phpR-cyp-F. RT-PCR amplification of the full length mRNAs of the control vector phpRF and the target vector phpR-cyp-F, using primers binding to the 5′ end of *renilla* and 3′ end of *firefly* open reading frames, yielded transcripts of the expected sizes (RF: 2587 nucleotides; R-cyp-F 2933 nucleotides) (Figure 5D, upper panel). Yet, while this observation verifies the generation of the full bicistronic mRNA, it does not rule out the presence of additional monocistronic *firefly* mRNAs. Therefore, we determined the expression of both *renilla* and *firefly* mRNAs separately in cells transfected with either phpRF control or phpR-cyp-F vectors. The assessment of *firefly* and *renilla* mRNAs revealed that the ratio of *firefly* to *renilla* mRNA remained unaltered upon insertion of the cyp24a1-5′UTR as compared to the empty vector control (Figure 5D, lower panel). This served as further evidence for the correct assignment of the IRES activity and ruled cryptic splicing and promoter activities out. To test if the cyp24a1 IRES activity, as determined using the bicistronic vector system, required a nuclear experience of the mRNA we next transfected *in vitro*-transcribed mRNA of the respective bicistronic vectors into MCF7 cells. In accordance with the transfection of plasmid DNA, *renilla* luciferase activities remained unaltered in the mRNA transfections of the control vs. the cyp24a1-containing bicistronic vector, and again, *firefly* luciferase activity was strongly enhanced upon intercistronic insertion of cyp24a1-5′UTR (Figure 5E). As the influence of both splicing and promoter events is restricted to the nuclear compartment, this experiment provided final proof for the presence of an IRES element within the 5′UTR of cyp24a1.

Conclusively, these results not only provide compelling evidence for the presence of an IRES element within the 5′UTR of cyp24a1 mRNA, but also validate the bicistronic reporter vector as a tool to characterize the regulation of the cyp24a1 IRES activity.

### IRES-dependent translation of cyp24a1 is activated by CM in a PI3K-dependent manner

**Figure 6:**
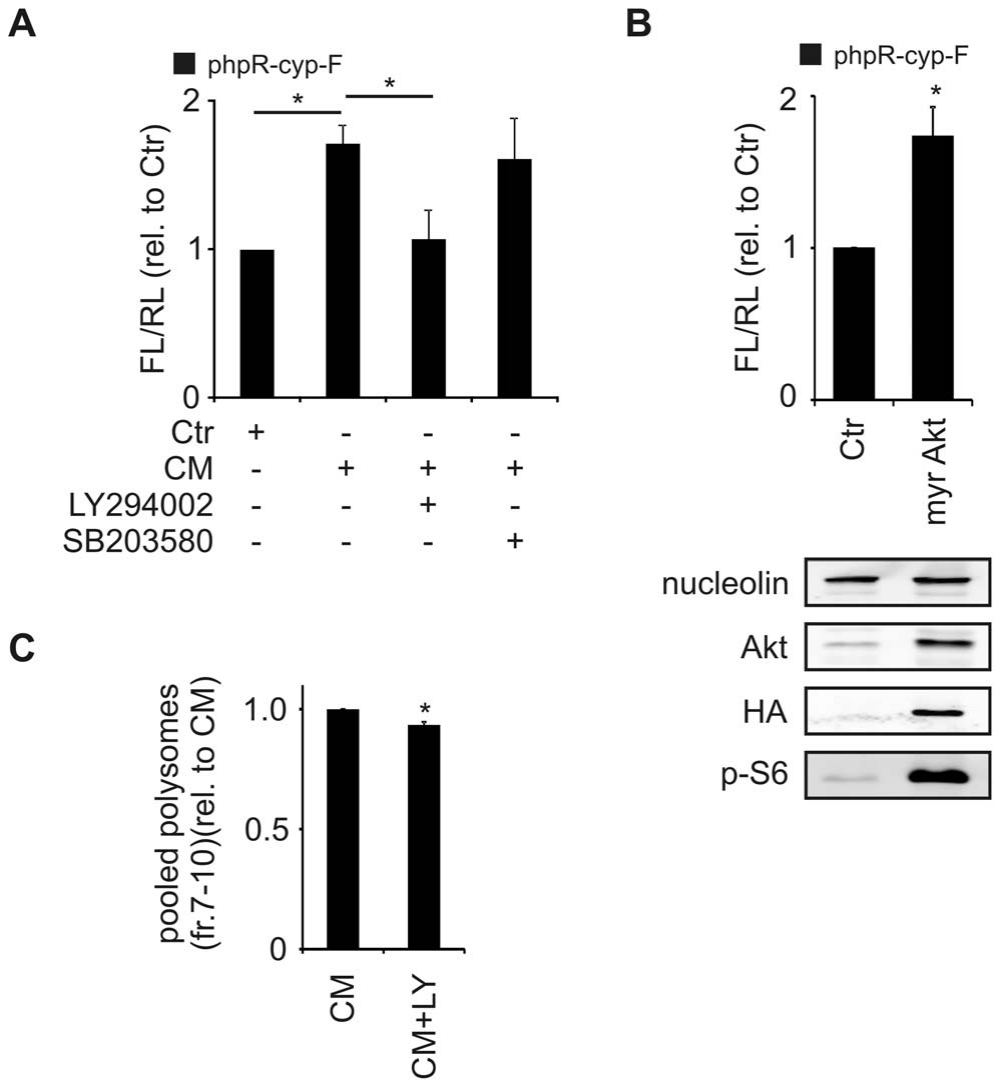
CM induces cyp24a1 IRES activity in an Akt-dependent manner. (A) MCF7 cells were transfected with phpR-cyp-F. 48 h after transfection cells were treated for 4 h with Ctr, CM, or CM supplemented with LY294002 [10 µM] or SB203580 [10 µM]. IRES activity was calculated as ratio of *firefly* to *renilla* luciferase activities and is given relative to Ctr. Data are presented as means ± SEM (n≥3, * p<0.05). (B) *(upper panel)* HEK293 cells overexpressing HA-tagged myr Akt were transfected with phpR-cyp-F. 48 h after transfection IRES activity was calculated as ratio of *firefly* to *renilla* luciferase activities and is given relative to control vector transfected cells. Data are presented as means ± SEM (n≥3, * p<0.05). *(lower panel)* HEK293 cells stably overexpressing HA-tagged myr Akt were serum starved for 48 h. Protein expression and S6-phosphorylation was determined by Western analysis. (C) MCF7 cells were treated for 4 h with CM or CM in combination with LY294002 [10 µM] followed by polysomal fractionation. Changes in cyp24a1 mRNA distribution were analyzed as described before. Data of pooled polysomal fractions (7–10) are presented as means ± SEM (n≥3, * p<0.05).

Finally, we aimed at determining if IRES-dependent cyp24a1 translation is responsive to CM. To this end we exposed MCF7 cells transfected with the cyp24a1 IRES reporter to Ctr and CM for 4 h. CM significantly enhanced the cyp24a1 IRES activity 1.71±0.13 fold compared to Ctr. This increase was comparable to the previously published activation of egr2 IRES activity by CM (1.5±0.18 fold) [16]. CM-induced cyp24a1 IRES activity was reduced by the PI3K inhibitor LY294002 [10 µM] to 1.07±0.20 relative to Ctr-treated cells, while inhibition of p38-MAPK by SB203580 [10 µM] was without effect (Figure 6A) in contrast to egr2 IRES activation [16]. To assess, if activation of the PI3K-Akt axis alone suffices to induce cyp24a1 IRES activity, we employed cells stably overexpressing constitutively active Akt (myr Akt) [17]. Overexpression of Akt and enhanced phosphorylation of the downstream target ribosomal protein S6 was verified using western analysis (Figure 6B). In line with the observed PI3K-dependency, overactivation of Akt enhanced cyp24a1 IRES activity (Figure 6B). To determine, if the altered IRES activity eventually affects translation efficiency of cyp24a1, we performed polysomal fractionation analysis of MCF7 cells treated with CM, compared to cells receiving CM in combination with the PI3K inhibitor LY294002. Indeed, inhibition of PI3K activity significantly reduced the polysomal abundance of cyp24a1 mRNA as compared to cells treated with CM alone (Figure 6C).

We conclude that IRES-dependent cyp24a1 translation is affected by PI3K. The observation that PI3K inhibition reduced total cyp24a1 translation in response to CM serves as another indicator that cyp24a1 translation is, at least in part, IRES-dependent.

## Discussion

In this study, we identify and characterize the translational regulation of cyp24a1. When MCF7 human mammary adenocarcinoma cells are exposed to an inflammatory microenvironment cyp24a1 mRNA shifts from monosomal to polysomal fractions. An IRES element within the 5′UTR of cyp24a1 mRNA, which is activated in a PI3K-Akt-dependent manner in response to inflammatory conditions, contributes to the enhanced translation of cyp24a1.

So far cyp24a1 expression was primarily characterized at the transcriptional level mediated by vitamin D_3_ (calcitriol) binding to the vitamin D receptor (VDR) [25], [26]. In addition, other nuclear receptors such as the pregnane X receptor [27] or the constitutive androstane receptor [28] influence cyp24a1 transcription. Both transcription factors facilitate transcription of cyp24a1, yet they inhibit vitamin D_3_-induced transcription by preventing the vitamin D_3_-mediated release of the repressive silencing mediator for retinoid and thyroid hormone receptors [29]. In addition, cyp24a1 expression might also be affected by DNA methylation [30]. Despite ample evidence for the transcriptional regulation of cyp24a1, little is known about post-transcriptional mechanisms affecting its expression. In the present study, we found that cyp24a1 expression can also be controlled at the level of translation. Specifically, we identified an IRES element within the cyp24a1 5′UTR, which was induced by inflammatory conditions (i.e. CM) and enhanced translation (Figure 2+6). As we have previously characterized egr2 translation to be regulated in an IRES-dependent manner in response to CM [16], IRES activation under inflammatory conditions appears to be a common event. The contribution of cryptic promoters, splicing, or read-through was experimentally excluded (Figure 5D+E). The notion of IRES-dependent cyp24a1 translation was further supported by the increased polysomal association of cyp24a1 mRNA under conditions where cap-dependent translation was repressed (Figure 4). This observation is in line with the concept that active PI3K-mTOR signaling enhances cap-dependent translation via inactivation of the 4E-BP [23], while IRES-dependent translation is commonly induced when cap-dependent translation is attenuated [31]. Interestingly, activation of the cyp24a1 IRES also required PI3K-Akt signaling under inflammatory conditions (Figure 6). Similarly, interleukin-3- and granulocyte macrophage colony-stimulating factor-induced, IRES-dependent c-myc translation in leukemic cells was insensitive to mTOR inhibition, despite being efficiently attenuated by the PI3K inhibitor LY294002 [32]. Along the same line, Petz et al. recently demonstrated that the IRES-activity of laminin b1 in response to PDGF was facilitated by active mitogen-activated protein kinase (MAPK) and PI3K signaling [33]. The authors proposed enhanced PI3K- and MAPK-dependent cytoplasmic accumulation of the La protein as the underlying mechanism. Thus, future work will identify which potential ITAFs are involved in the IRES activation of cyp24a1.

Functionally, cyp24a1 is induced by vitamin D_3_, and at the same time plays an important role in its metabolic inactivation. Specifically, cyp24a1 is transcriptionally induced by the transcription factor VDR. VDR enhances the transcription of a set of more than 900 vitamin D_3_-induced genes. Importantly, cyp24a1, i.e. 1,25-dihydroxyvitamin D_3_ 24-hydroxylase, is the prime metabolizing enzyme to inactivate vitamin D_3_. Thus, increasing levels of cyp24a1 provide an efficient negative feedback loop to terminate vitamin D_3_ actions [34]. Since vitamin D_3_ is used as an adjuvant tumor therapeutic agent, this negative feedback loop may contribute to the development of resistances against vitamin D_3_. Yet, cyp24a1 is not only associated with a poor response to vitamin D_3_-based therapeutics, it rather appears to be overexpressed in various tumor types [35]–[37]. Along these lines, Horvath et al. recently proposed cyp24a1 as a novel biomarker for colon tumorigenesis [38]. They found highest cyp24a1 mRNA expression in benign colorectal lesions compared to normal colonic tissue and adenocarcinomas, while cyp24a1 protein expression was highest in advanced adenocarcinomas as compared to early stages or normal tissue. This phenotype strongly supports an additional, post-transcriptional level of cyp24a1 regulation. As colon tumors are commonly associated with inflammatory conditions, our findings of enhanced inflammation-induced, IRES-dependent translation might serve as a rational explanation for the observed discrepancy between mRNA and protein amount. In line, we have previously shown that the inflammatory microenvironment as generated by activated monocyte-derived macrophages, indeed elicits pro-tumorigenic responses such as transformation and invasion [19].

Taken together, we provide evidence for a novel mechanism of cyp24a1 regulation via PI3K-facilitated, IRES-dependent translation under pro-inflammatory conditions. Further studies are required to elucidate the functional consequences of altered cyp24a1 translation but also to determine changes in cyp24a1 translation in other tumor models. Importantly, the regulation of cyp24a1 translation, specifically via an alternative mode of initiation, might open novel therapeutic options to sensitize tumors to vitamin D_3_ treatment or to overcome resistances.

## References

[1] Silvera D, Formenti SC, Schneider RJ (2010) Translational control in cancer. Nat Rev Cancer 10: 254–266. 20332778 10.1038/nrc2824

[2] Sonenberg N, Hinnebusch AG (2009) Regulation of translation initiation in eukaryotes: mechanisms and biological targets. Cell 136: 731–745. 19239892 10.1016/j.cell.2009.01.042 PMC3610329

[3] Guertin DA, Sabatini DM (2007) Defining the role of mTOR in cancer. Cancer Cell 12: 9–22. 17613433 10.1016/j.ccr.2007.05.008

[4] Pause A, Belsham GJ, Gingras AC, Donze O, Lin TA, et al (1994) Insulin-dependent stimulation of protein synthesis by phosphorylation of a regulator of 5′-cap function. Nature 371: 762–767. 7935836 10.1038/371762a0

[5] Mamane Y, Petroulakis E, LeBacquer O, Sonenberg N (2006) mTOR, translation initiation and cancer. Oncogene 25: 6416–6422. 17041626 10.1038/sj.onc.1209888

[6] Averous J, Fonseca BD, Proud CG (2008) Regulation of cyclin D1 expression by mTORC1 signaling requires eukaryotic initiation factor 4E-binding protein 1. Oncogene 27: 1106–1113. 17724476 10.1038/sj.onc.1210715

[7] Blagden SP, Willis AE (2011) The biological and therapeutic relevance of mRNA translation in cancer. Nat Rev Clin Oncol 8: 280–291. 21364523 10.1038/nrclinonc.2011.16

[8] Faivre S, Kroemer G, Raymond E (2006) Current development of mTOR inhibitors as anticancer agents. Nat Rev Drug Discov 5: 671–688. 16883305 10.1038/nrd2062

[9] Holcik M (2004) Targeting translation for treatment of cancer–a novel role for IRES? Curr Cancer Drug Targets 4: 299–311. 15134536 10.2174/1568009043333005

[10] Spriggs KA, Stoneley M, Bushell M, Willis AE (2008) Re-programming of translation following cell stress allows IRES-mediated translation to predominate. Biol Cell 100: 27–38. 18072942 10.1042/BC20070098

[11] Lang KJ, Kappel A, Goodall GJ (2002) Hypoxia-inducible factor-1alpha mRNA contains an internal ribosome entry site that allows efficient translation during normoxia and hypoxia. Mol Biol Cell 13: 1792–1801. 12006670 10.1091/mbc.02-02-0017 PMC111144

[12] Lewis SM, Holcik M (2005) IRES in distress: translational regulation of the inhibitor of apoptosis proteins XIAP and HIAP2 during cell stress. Cell Death Differ 12: 547–553. 15818406 10.1038/sj.cdd.4401602

[13] Sherrill KW, Byrd MP, Van Eden ME, Lloyd RE (2004) BCL-2 translation is mediated via internal ribosome entry during cell stress. J Biol Chem 279: 29066–29074. 15123638 10.1074/jbc.M402727200

[14] Spriggs KA, Mitchell SA, Willis AE (2005) Investigation of interactions of polypyrimidine tract-binding protein with artificial internal ribosome entry segments. Biochem Soc Trans 33: 1483–1486. 16246151 10.1042/BST0331483

[15] Dhamija S, Kuehne N, Winzen R, Doerrie A, Dittrich-Breiholz O, et al (2011) Interleukin-1 Activates Synthesis of Interleukin-6 by Interfering with a KH-type Splicing Regulatory Protein (KSRP)-dependent Translational Silencing Mechanism. J Biol Chem 286: 33279–33288. 21795706 10.1074/jbc.M111.264754 PMC3190945

[16] Rübsamen D, Blees JS, Schulz K, Doring C, Hansmann ML, et al (2012) IRES-dependent translation of egr2 is induced under inflammatory conditions. RNA 18: 1910–1920. 22915601 10.1261/rna.033019.112 PMC3446713

[17] Schmid T, Jansen AP, Baker AR, Hegamyer G, Hagan JP, et al (2008) Translation inhibitor Pdcd4 is targeted for degradation during tumor promotion. Cancer Res 68: 1254–1260. 18296647 10.1158/0008-5472.CAN-07-1719

[18] Coldwell MJ, Mitchell SA, Stoneley M, MacFarlane M, Willis AE (2000) Initiation of Apaf-1 translation by internal ribosome entry. Oncogene 19: 899–905. 10702798 10.1038/sj.onc.1203407

[19] Schmid T, Bajer MM, Blees JS, Eifler LK, Milke L, et al (2011) Inflammation-induced loss of Pdcd4 is mediated by phosphorylation-dependent degradation. Carcinogenesis 32: 1427–1433. 21771721 10.1093/carcin/bgr131 PMC3179419

[20] Yasuda M, Schmid T, Rübsamen D, Colburn NH, Irie K, et al (2010) Downregulation of programmed cell death 4 by inflammatory conditions contributes to the generation of the tumor promoting microenvironment. Mol Carcinog 49: 837–848. 20607724 10.1002/mc.20660 PMC3472367

[21] Gruber AR, Lorenz R, Bernhart SH, Neubock R, Hofacker IL (2008) The Vienna RNA websuite. Nucleic Acids Res 36: W70–74. 18424795 10.1093/nar/gkn188 PMC2447809

[22] van der Velden AW, Thomas AA (1999) The role of the 5′ untranslated region of an mRNA in translation regulation during development. Int J Biochem Cell Biol 31: 87–106. 10216946 10.1016/s1357-2725(98)00134-4

[23] Thoreen CC, Chantranupong L, Keys HR, Wang T, Gray NS, et al (2012) A unifying model for mTORC1-mediated regulation of mRNA translation. Nature 485: 109–113. 22552098 10.1038/nature11083 PMC3347774

[24] Van Eden ME, Byrd MP, Sherrill KW, Lloyd RE (2004) Demonstrating internal ribosome entry sites in eukaryotic mRNAs using stringent RNA test procedures. RNA 10: 720–730. 15037781 10.1261/rna.5225204 PMC1370562

[25] Chen KS, DeLuca HF (1995) Cloning of the human 1 alpha,25-dihydroxyvitamin D-3 24-hydroxylase gene promoter and identification of two vitamin D-responsive elements. Biochim Biophys Acta 1263: 1–9. 7632726 10.1016/0167-4781(95)00060-t

[26] Horvath E, Lakatos P, Balla B, Kosa JP, Tobias B, et al (2012) Marked increase of CYP24A1 mRNA level in hepatocellular carcinoma cell lines following vitamin D administration. Anticancer Res 32: 4791–4796. 23155244

[27] Pascussi JM, Robert A, Nguyen M, Walrant-Debray O, Garabedian M, et al (2005) Possible involvement of pregnane X receptor-enhanced CYP24 expression in drug-induced osteomalacia. J Clin Invest 115: 177–186. 15630458 10.1172/JCI21867 PMC539191

[28] Moreau A, Maurel P, Vilarem MJ, Pascussi JM (2007) Constitutive androstane receptor-vitamin D receptor crosstalk: consequence on CYP24 gene expression. Biochem Biophys Res Commun 360: 76–82. 17585873 10.1016/j.bbrc.2007.06.003

[29] Konno Y, Kodama S, Moore R, Kamiya N, Negishi M (2009) Nuclear xenobiotic receptor pregnane X receptor locks corepressor silencing mediator for retinoid and thyroid hormone receptors (SMRT) onto the CYP24A1 promoter to attenuate vitamin D3 activation. Mol Pharmacol 75: 265–271. 18981260 10.1124/mol.108.051904 PMC2684893

[30] Novakovic B, Sibson M, Ng HK, Manuelpillai U, Rakyan V, et al (2009) Placenta-specific methylation of the vitamin D 24-hydroxylase gene: implications for feedback autoregulation of active vitamin D levels at the fetomaternal interface. J Biol Chem 284: 14838–14848. 19237542 10.1074/jbc.M809542200 PMC2685665

[31] Svitkin YV, Herdy B, Costa-Mattioli M, Gingras AC, Raught B, et al (2005) Eukaryotic translation initiation factor 4E availability controls the switch between cap-dependent and internal ribosomal entry site-mediated translation. Mol Cell Biol 25: 10556–10565. 16287867 10.1128/MCB.25.23.10556-10565.2005 PMC1291233

[32] Kobayashi N, Saeki K, Yuo A (2003) Granulocyte-macrophage colony-stimulating factor and interleukin-3 induce cell cycle progression through the synthesis of c-Myc protein by internal ribosome entry site-mediated translation via phosphatidylinositol 3-kinase pathway in human factor-dependent leukemic cells. Blood 102: 3186–3195. 12855588 10.1182/blood-2003-02-0567

[33] Petz M, Them NC, Huber H, Mikulits W (2012) PDGF enhances IRES-mediated translation of Laminin B1 by cytoplasmic accumulation of La during epithelial to mesenchymal transition. Nucleic Acids Res 40: 9738–9749. 22904067 10.1093/nar/gks760 PMC3479205

[34] Schuster I (2011) Cytochromes P450 are essential players in the vitamin D signaling system. Biochim Biophys Acta 1814: 186–199. 20619365 10.1016/j.bbapap.2010.06.022

[35] Hobaus J, Hummel DM, Thiem U, Fetahu IS, Aggarwal A, et al. (2013) Increased copy-number and not DNA hypomethylation causes overexpression of the candidate proto-oncogene CYP24A1 in colorectal cancer. Int J Cancer. 10.1002/ijc.28143 PMC3807607 23463632

[36] Lopes N, Sousa B, Martins D, Gomes M, Vieira D, et al (2010) Alterations in Vitamin D signalling and metabolic pathways in breast cancer progression: a study of VDR, CYP27B1 and CYP24A1 expression in benign and malignant breast lesions. BMC Cancer 10: 483. 20831823 10.1186/1471-2407-10-483 PMC2945944

[37] Balla B, Kosa JP, Tobias B, Halaszlaki C, Takacs I, et al (2011) Marked increase in CYP24A1 gene expression in human papillary thyroid cancer. Thyroid 21: 459–460. 21385079 10.1089/thy.2010.0420

[38] Horvath HC, Lakatos P, Kosa JP, Bacsi K, Borka K, et al (2010) The candidate oncogene CYP24A1: A potential biomarker for colorectal tumorigenesis. J Histochem Cytochem 58: 277–285. 19901270 10.1369/jhc.2009.954339 PMC2825493

